# Preparation and Hypoglycemic Activity of Enzymatically Hydrolyzed Peptides From the Swim Bladder of *Pangasius bocourti*

**DOI:** 10.1002/fsn3.72061

**Published:** 2026-07-01

**Authors:** Ju Yang, Xu Qiu, Huan Zhang, Kefeng Wu, Yushuang Li, Guangxin Xu, Jianlin He, Junde Chen, Xixiang Tang

**Affiliations:** ^1^ Marine Biomedical Research Institute Guangdong Medical University Zhanjiang Guangdong China; ^2^ Third Institute of Oceanography Ministry of Natural Resources Xiamen Fujian China

**Keywords:** enzymatic hydrolysis, hypoglycemic activity, nano‐LC–MS/MS, *Pangasius bocourti*, swim bladder peptides

## Abstract

The swim bladder of *Pangasius bocourti* is an underutilized fishery byproduct with high protein content, yet its potential as a source of hypoglycemic peptides has not been systematically investigated. This study aimed to prepare and characterize bioactive peptides from *P. bocourti* swim bladder (PBSB) through integrated enzymatic hydrolysis optimization, peptidomic analysis, and in vivo evaluation. Five proteases were comparatively assessed, and alkaline protease was identified as the optimal enzyme based on degree of hydrolysis and α‐glucosidase inhibitory activity. Under the optimized conditions (pH 9.5, enzyme dosage 2%, 50°C, 4 h), the obtained peptides (PBSBPs) exhibited notable α‐glucosidase inhibitory activity (67.90% ± 0.26% at 5 mg/mL). Nano‐LC–MS/MS analysis identified 101 peptides, of which 91.09% had molecular weights below 1500 Da. The peptide sequences were enriched in hydrophobic and hypoglycemia‐associated amino acid residues, and 58 peptides were predicted to possess α‐glucosidase inhibitory potential. Furthermore, PBSBPs significantly improved glucose tolerance, reduced serum triglyceride and total cholesterol levels, and alleviated hepatic lipid accumulation and pancreatic islet injury in diabetic mice. This study integrates process optimization, peptide profiling, and biological validation, providing a systematic strategy for the development of functional peptides from aquatic byproducts. These findings highlight the potential application of PBSBPs as natural antidiabetic functional ingredients and contribute to the high‐value utilization of fishery processing byproducts.

## Graphical abstract

Fish swim bladder is a low‐value byproduct from *Pangasius bocourti* processing. Herein, we successfully prepared hypoglycemic bioactive peptides from *P. bocourti* swim bladder (PBSBPs) via optimized alkaline protease hydrolysis. Peptidomic analysis revealed abundant low‐molecular‐weight peptides rich in hydrophobic amino acids with predicted α‐glucosidase inhibitory capacity. In vivo experiments confirmed that PBSBPs relieve hyperglycemia, regulate blood lipid metabolism, protect pancreatic islets, and reduce hepatic lipid deposition in diabetic mice. This work offers a feasible route to convert aquatic processing waste into natural anti‐diabetic functional peptides and realizes high‐value reutilization of fishery byproducts.

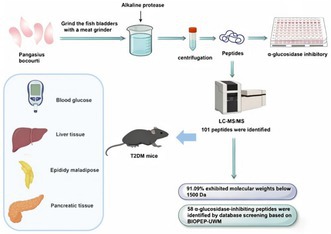

## 1 Introduction

Diabetes mellitus is a prevalent chronic metabolic disorder associated with multiple complications, including retinopathy, neuropathy, and nephropathy, which severely impair human health and quality of life (G.‐L. Chen et al. 2020; Hong et al. 2020). The global prevalence of diabetes among adults aged 20–79 years is projected to increase from 10.5% to 12.2% between 2021 and 2045, accompanied by continuously rising healthcare expenditures (Sun et al. 2022). Although current hypoglycemic agents such as metformin and insulin are widely used in clinical practice, their long‐term application is often associated with gastrointestinal side effects, risk of hypoglycemia, and high economic burden (Kelly et al. 2023). Therefore, the development of safe, effective, and sustainable hypoglycemic agents from natural resources has attracted increasing attention.

In recent years, food‐derived bioactive peptides have emerged as promising candidates for glycemic regulation owing to their high biocompatibility, diverse biological activities, and relatively low toxicity. Previous studies have demonstrated that bioactive peptides can regulate glucose metabolism through multiple mechanisms, including inhibition of carbohydrate‐hydrolyzing enzymes such as α‐amylase and α‐glucosidase, as well as dipeptidyl peptidase‐IV (DPP‐IV). In addition, many peptides exhibit antioxidant and anti‐inflammatory activities, which may further contribute to the alleviation of diabetic complications. For example, peptides derived from casein hydrolysates exhibited stronger α‐amylase inhibitory activity than metformin in vitro (Aziz et al. 2025), while potent ACE‐ and DPP‐IV‐inhibitory peptides have been identified from *Pyropia vietnamensis* proteins (Basri et al. 2025). Hydrolysates prepared from defatted Antarctic krill ( *Euphausia superba* ) powder also showed significant α‐glucosidase inhibitory activity (Zheng et al. 2024). Similarly, bioactive peptides derived from goat milk whey proteins, hot‐pressed peanut meal proteins, and soft‐shelled turtle eggs have demonstrated considerable hypoglycemic potential (Du et al. 2022; Li et al. 2022; Yang et al. 2023). These findings highlight the considerable potential of food‐derived peptides as functional ingredients for diabetes management. Among the available preparation strategies, enzymatic hydrolysis is widely used due to its mild reaction conditions, high specificity, environmental friendliness, and ability to generate diverse bioactive peptide sequences.

Fish swim bladders are collagen‐rich protein resources with low lipid content and have attracted increasing attention as potential sources of bioactive peptides. Previous studies have reported that swim bladder‐derived hydrolysates possess various biological activities, including hypoglycemic (Hong et al. 2020), antioxidant (Chen et al. 2019; Zeng et al. 2024), antihypertensive (Dong and Dai 2023; Hu et al. 2023), and anti‐aging effects (Lu et al. 2024; Zhihui et al. 2024). *Pangasius bocourti* , a commercially important freshwater fish widely cultivated in Southeast Asia, represents one of the most important species in the global freshwater aquaculture and processing industry (Yang et al. 2023; Zhang et al. 2022). Owing to its high fillet yield (approximately 40%), rapid growth, and favorable processing characteristics, *P. bocourti* has become an important raw material for large‐scale aquatic food processing and international trade (Boyd et al. 2022; Dhar et al. 2021). However, extensive industrial processing of *P. bocourti* also generates substantial quantities of by‐products, including fish heads, viscera, bones, skin, and swim bladders. Previous studies have reported that by‐products may account for up to 58.80% of the total fish mass during processing, indicating considerable underutilization of protein‐rich resources (Vu et al. 2024). Among these by‐products, swim bladder is a collagen‐rich tissue with promising potential for the preparation of functional peptides. Nevertheless, the preparation, peptide characterization, and hypoglycemic activity of peptides derived from *P. bocourti* swim bladder have not yet been systematically investigated.

Therefore, the present study aimed to optimize the enzymatic hydrolysis conditions for preparing bioactive peptides from *P. bocourti* swim bladder (PBSB) and evaluate their α‐glucosidase inhibitory activity in vitro. Furthermore, nano‐liquid chromatography–tandem mass spectrometry (nano‐LC–MS/MS) was employed to characterize the peptide composition of PBSB peptides (PBSBPs), while PeptideRanker and BIOPEP databases were used to predict the hypoglycemic potential of the identified peptides. In addition, the in vivo hypoglycemic effects of PBSBPs were evaluated using diabetic mouse models. This study provides a theoretical basis for the development of natural hypoglycemic peptides from aquatic by‐products and contributes to the high‐value utilization of freshwater fish processing wastes.

## 2 Materials and Methods

### 2.1 Materials

PBSBs were procured from Beihai Kuanli Aquatic Products Co. Ltd. (Guangxi, China). C57BL/6 mice (18–21 g) were obtained from Guangdong Yaokang Biotechnology Co. Ltd. (Guangdong, China; Certificate number: SCXK (Yue) 2020–0054). Alkaline protease (200,000 U/g), animal protease (100,000 U/g), neutral protease (200,000 U/g), papain (200,000 U/g), and flavor protease (20,000 U/g) were purchased from Nanning Pangbo Biological Engineering Co. Ltd. (Guangxi, China). α‐Glucosidase was purchased from Shanghai Yuanye Biotechnology Co. Ltd. (Shanghai, China). All reagents used in this study were of analytical grade.

### 2.2 Preparation of PBSBPs

PBSBs were first trimmed to remove surface adipose tissue, followed by washing and draining. The cleaned PBSBs were then minced into small fragments and homogenized. Purified water was added to the homogenate at a solid‐to‐liquid ratio of 1:20 (w/v), and the mixture was heated at 90°C for 1 h with continuous stirring to facilitate protein extraction. Subsequently, the pH and temperature of the reaction system were adjusted according to the designated enzymatic hydrolysis conditions, and the corresponding protease was added. Because the commercial proteases used in this study possessed different declared enzyme activity units, each enzyme was applied according to the manufacturer‐recommended dosage conditions commonly adopted in food protein hydrolysis studies. Therefore, the protease screening experiment was intended as a preliminary comparative evaluation under practical hydrolysis conditions rather than a strict comparison under normalized catalytic activity.

After hydrolysis, the reaction mixture was heated at 90°C for 10 min to inactivate the enzyme. The hydrolysate was then centrifuged at 9000 rpm for 10 min using a refrigerated centrifuge, and the supernatant was collected and freeze‐dried for subsequent analyses.

### 2.3 Determination of Moisture and Protein Contents of PBSB

The moisture and protein contents of PBSB were determined prior to enzymatic hydrolysis. Moisture content was measured using the oven‐drying method. Briefly, PBSB samples were dried in an oven at 105°C until constant weight was achieved, and moisture content was calculated based on the mass difference before and after drying. Protein content was determined using the Kjeldahl method according to a previously reported procedure (Rizvi et al. 2022), with a nitrogen‐to‐protein conversion factor of 6.25.

### 2.4 Determination of Hydrolysis Degree (DH)

The degree of hydrolysis (DH) during enzymatic hydrolysis of PBSB was determined using the pH‐stat method according to previous studies (Mirzakhani et al. 2018; Yolandani et al. 2023). During hydrolysis, the pH of the reaction system was continuously maintained at the designated value by addition of 1 mol/L NaOH solution, and the volume of NaOH consumed was recorded. After hydrolysis, the reaction mixture was heated at 90°C for 10 min to inactivate the enzyme. DH was then calculated based on the volume and concentration of consumed NaOH according to Equation (1):

(1) $$\mathrm{DH}=\frac{B\times N_{b}}{M_{p}\times \alpha \times H_{\mathrm{tot}}}\times 100\%$$

where DH represents the degree of hydrolysis; *B* is the volume of NaOH consumed (mL); *N*_*b*_ is the concentration of NaOH (mol/L); *M*_*P*_ is the mass of substrate protein (g); is the average degree of dissociation of α‐amino groups, which is related to pH and temperature; and *H*_*tot*_ represents the total number of peptide bonds in the substrate protein. For fish proteins, the *H*_*tot*_ value was set as 8.6 meq/g protein according to previous reports (Rivero‐Pino et al. 2020).

### 2.5 Determination of the α‐Glucosidase Inhibitory Activity

The α‐glucosidase inhibitory activity of PBSBPs was determined according to previously reported methods with minor modifications (Yu et al. 2024; Zheng et al. 2024). Briefly, for the sample group, 50 μL of α‐glucosidase solution (0.5 U/mL) was mixed with 25 μL of PBSBPs solution (5 mg/mL) and pre‐incubated at 37°C for 10 min. Subsequently, 25 μL of p‐nitrophenyl‐α‐D‐glucopyranoside (PNPG) solution (2 mmol/L) was added, and the reaction mixture was further incubated at 37°C for 15 min. The reaction was terminated by adding 100 μL of Na_2_CO_3_ solution (0.2 mol/L).

For the sample blank group, 50 μL of phosphate‐buffered saline (PBS, 0.1 mol/L, pH 6.8) was added instead of the α‐glucosidase solution, while all other procedures remained identical to those of the sample group. For the control group, 25 μL of PBS and 50 μL of α‐glucosidase solution were added, followed by the same procedures described above. In the control blank group, 75 μL of PBS was added instead of both sample and enzyme solutions, while the remaining procedures were unchanged.

After completion of the reaction, absorbance at 405 nm was measured using a microplate reader. The α‐glucosidase inhibitory activity was calculated according to Equation (2):

(2) $$\alpha -\text{glucosidase inhibition}\;\left(\%\right)=\left(1-\frac{C-D}{A-B}\right)\times 100\%$$

where *A*, *B*, *C*, and *D* represent the absorbance values of the control group, control blank group, sample group, and sample blank group, respectively.

### 2.6 Optimization of Enzymatic Hydrolysis Process

#### 2.6.1 Screening of Protease

Neutral protease, flavor protease, animal protease, alkaline protease, and papain were employed to hydrolyze PBSB. The optimal protease was selected based on both the degree of hydrolysis (DH) and α‐glucosidase inhibitory activity of the resulting hydrolysates. DH was used to evaluate hydrolysis efficiency, whereas α‐glucosidase inhibitory activity was employed to assess the potential hypoglycemic activity of the generated peptides. The enzymatic hydrolysis conditions for each protease were selected according to their respective optimal catalytic conditions reported in previous studies (Cai et al. 2024; Li et al. 2023; Yin et al. 2021), as summarized in Table 1.

**TABLE 1:** The enzymatic hydrolysis conditions of enzymes.

Type of enzyme	Temperature/°C	pH	Time/hours	Enzyme dosage
Alkaline protease	55	8.5	4	1%
Flavor protease	50	7.0	4	1%
Animal protease	50	7.0	4	1%
Papain	50	7.0	4	1%
Neutral protease	50	7.0	4	1%

#### 2.6.2 Single‐Factor Experiments

Based on the preliminary protease screening experiments, alkaline protease was selected for further study. To investigate the effects of different hydrolysis parameters on the DH and α‐glucosidase inhibition rate, single‐factor experiments were conducted by varying one parameter while keeping the others constant. Specifically, hydrolysis temperatures were set at 40°C, 45°C, 50°C, 55°C, and 60°C; hydrolysis pH values were adjusted to 7.5, 8.5, 9.5, 10.5, and 11.5; hydrolysis times were set at 1, 2, 3, 4, 5, and 6 h; enzyme dosages were adjusted to 1%, 2%, 3%, 4%, and 5% (w/w); and solid‐to‐liquid ratios were set at 1:10, 1:20, 1:30, 1:40, and 1:50 (g/mL). The purpose of these experiments was to preliminarily clarify the relationship between hydrolysis conditions, DH, and α‐glucosidase inhibitory activity, thereby providing a basis for subsequent orthogonal optimization.

#### 2.6.3 Orthogonal Experiment

In this study, based on the results of the single‐factor experiments, the α‐glucosidase inhibition rate was used as the evaluation index. Four key factors, namely hydrolysis temperature, hydrolysis pH, hydrolysis time, and enzyme dosage, were selected to design a four‐factor, three‐level orthogonal experiment. The aim was to determine the optimal enzymatic hydrolysis process for PBSB. The specific experimental conditions are presented in Table 2. Orthogonal optimization was employed to comprehensively evaluate the interactions among major hydrolysis parameters and to obtain hydrolysates with enhanced α‐glucosidase inhibitory activity under relatively efficient processing conditions.

**TABLE 2:** Orthogonal design table.

Experiment No.	(A) Temperature/°C	(B) pH	(C) Time/hours	(D) Enzyme dosage/%
1	50	9.5	3	2
2	50	10.5	4	3
3	50	11.5	5	4
4	55	9.5	4	4
5	55	10.5	5	2
6	55	11.5	3	3
7	60	9.5	5	3
8	60	10.5	3	4
9	60	11.5	4	2

### 2.7 Nano‐LC–MS/MS

Since the biological activity of protein hydrolysates is closely associated with peptide composition and sequence characteristics, Nano‐LC–MS/MS was employed to identify the major peptide components in PBSBPs and to further explore the possible structural basis of their hypoglycemic activity. The PBSBPs sample was desalted, followed by vacuum centrifugal concentration and drying. The dried sample was redissolved in 0.1% formic acid in water and separated using a nano‐liquid chromatography system (UltiMate 3000 RSLCnano, Thermo Fisher Scientific, Waltham, MA, USA). The chromatographic column was PepMap C18 (75 μm × 25 cm, 2 μm, Thermo Fisher Scientific, Waltham, MA, USA). Mobile phase A was 0.1% formic acid in water, while mobile phase B was 0.1% formic acid in acetonitrile. The elution gradient was as follows: 0–50 min, 5%–38% B; 50–52 min, 38%–95% B; and 52–60 min, 95% B. The flow rate was maintained at 300 nL/min throughout the analysis.

Mass spectrometry analysis was conducted using a Q Exactive mass spectrometer (Thermo Fisher Scientific, Waltham, MA, USA) operated in data‐dependent acquisition mode. The precursor ion scan range was 300–1500 m/z. For MS1, the resolution was 70,000 (m/z 200), the AGC target was 3e6, and the maximum ion injection time was 100 ms. For MS2, higher‐energy collisional dissociation was applied with a normalized collision energy of 28%, a resolution of 17,500, an AGC target of 1e5, a maximum injection time of 50 ms, and a dynamic exclusion time of 25 s.

Data analysis was conducted using PEAKS Studio 8.5 (Bioinformatics Solutions Inc., Waterloo, Canada), with the database set as UniProt‐*Pangasianodon hypophthalmus*‐UP000327468. Search parameters included a mass tolerance of 10 ppm for MS1 and 0.03 Da for MS2. Additionally, variable modifications such as protein N‐terminal acetylation, asparagine/glutamine deamidation, and methionine oxidation were considered. After screening, peptides with −10lgP > 60 were selected for further analysis.

### 2.8 Prediction of the Activity and Analysis of Peptides

The bioactivity of the peptides was analyzed using the PeptideRanker tool (https://distilldeep.ucd.ie/PeptideRanker/). PeptideRanker was used to preliminarily evaluate the probability of peptide bioactivity based on peptide sequence features, thereby facilitating the screening of potentially functional peptides from the complex hydrolysate system.

The α‐glucosidase inhibitory activity of the peptides was predicted using the BIOPEP‐UWM database (https://biochemia.uwm.edu.pl/biopep/start_biopep.php). BIOPEP‐UWM analysis was further performed to identify peptide fragments with reported or predicted α‐glucosidase inhibitory potential and to establish correlations between peptide structural characteristics and hypoglycemic activity.

The isoelectric point (pI) of the peptides was determined using the Expasy ProtParam tool (https://web.expasy.org/protparam).

### 2.9 In Vivo Activity Assays

#### 2.9.1 Experimental Design in Animal Studies

Eight‐week‐old male C57BL/6 mice were selected for the experiment. These mice were housed in a controlled environment with regulated temperature, humidity, and a 12 light/12 h dark cycle. A total of 50 mice were divided into five groups: one normal control group (NC) and four experimental groups. The NC group was fed a control diet containing 10% kcal from fat (D12450J, Research Diets, New Brunswick, NJ, USA). The other 40 mice were fed a high‐fat diet (60% kcal from fat, D12492, Research Diets) for 12 weeks to induce a diabetes mellitus model (Melkani et al. 2025).

After the model was established, these 40 diabetic mice were randomly divided into four groups: a model group (Model) maintained on the high‐fat diet, and three PBSBPs‐treated groups, including a low‐dose group (PBSBPs (L), 50), a middle‐dose group (PBSBPs (M), 100), and a high‐dose group (PBSBPs (H), 200 mg/kg body weight/day) (Han et al. 2023), while still being maintained on the high‐fat diet. PBSBPs were dissolved in sterile saline and administered once daily by oral gavage. The treatment lasted for 4 weeks. The dose design was based on previous studies investigating the hypoglycemic activity of food‐derived bioactive peptides in diabetic mouse models.

#### 2.9.2 Oral Glucose Tolerance Test (OGTT)

The mice were fasted for 6 h with free access to water. Following the fasting period, blood samples were collected from the tail vein, and fasting blood glucose levels were measured using a glucometer (ACON, Hangzhou, China). Subsequently, a glucose solution was administered to the mice by gavage at 2 g/kg body weight (Andrikopoulos et al. 2008). Blood glucose levels were then measured at 30 and 120 min post‐glucose administration using a glucometer. The area under the curve (AUC) of the OGTT was calculated to evaluate glucose tolerance and the in vivo hypoglycemic effect of PBSBPs.

#### 2.9.3 The Determination of Blood Lipids in Mice

Blood samples were collected from the retro‐orbital sinus for serum separation. The levels of total cholesterol (TC), triglycerides (TG), high‐density lipoprotein cholesterol (HDL‐C), and low‐density lipoprotein cholesterol (LDL‐C) in serum samples were analyzed using a veterinary biochemical analyzer (BS‐240Vet, Mindray, Shenzhen, China). Blood lipid parameters were determined to further assess the effects of PBSBPs on lipid metabolism disorders associated with diabetes mellitus.

#### 2.9.4 Histological Analysis

After the mice were sacrificed, the liver, pancreas, and epididymal adipose tissues were immediately excised and fixed in 10% formalin. Following fixation, the samples were dehydrated using a tissue dehydrator (Junjie, Wuhan, China), embedded in paraffin with an embedding machine (Junjie, Wuhan, China), and sectioned into thin slices using a pathological microtome (Leica, Shanghai, China). After sectioning, the slices were flattened with a tissue floater (Kedi, Zhejiang, China). These sections were then stained with hematoxylin and eosin (H&E). Subsequently, digital images of the stained tissue sections were captured using a microscope (Nikon Eclipse Ci‐L, Nikon, Tokyo, Japan) for further analysis. Histological examination was performed to evaluate tissue pathological changes and to further verify the protective effects of PBSBPs against diabetes‐associated hepatic, adipose, and pancreatic injuries.

### 2.10 Statistical Analysis

In this study, all experiments were repeated three times. The experimental data were analyzed by one‐way ANOVA combined with Duncan's multiple range test using SPSS 27 software (IBM Corp., Armonk, NY, USA) to determine the differences between groups (*p* < 0.05). Data were expressed as mean ± SD. Graphs were generated using Origin 24.0 (OriginLab Corporation, Northampton, MA, USA) and GraphPad Prism 9.5 (GraphPad Software, San Diego, CA, USA).

## 3 Results and Discussion

### 3.1 Optimization of Enzymatic Hydrolysis Process

#### 3.1.1 The Comparative Analysis on the Hydrolysis Efficiency of Different Proteases

**FIGURE 1:**
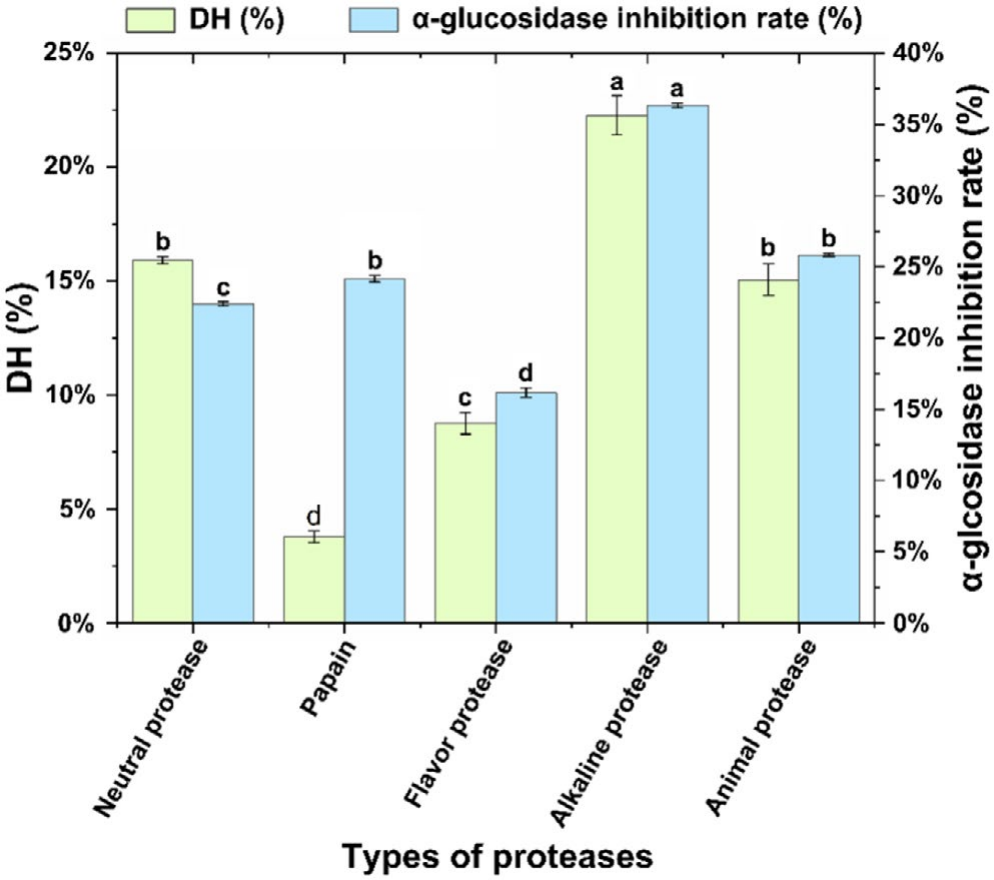
Effects of various proteases on the DH of PBSB and the inhibitory activity of the hydrolysates on α‐glucosidase. Values are presented as mean ± SD. Different lowercase letters indicate significant differences (*p* < 0.05, *n* = 3). DH, hydrolysis degree.

In this study, five different proteases—alkaline protease, papain, animal protease, neutral protease, and flavor protease—were employed to hydrolyze PBSB. The DH and α‐glucosidase inhibitory activities of the resulting hydrolysates are illustrated in Figure 1. The ranking of the DH values obtained using these proteases was as follows: alkaline protease> neutral protease > animal protease > flavor protease > papain. Under the tested hydrolysis conditions, hydrolysates prepared using alkaline protease exhibited the highest α‐glucosidase inhibitory activity among all tested groups. Its superior hydrolysis performance is consistent with previous studies on swim bladder protein hydrolysis (Dong and Dai 2023), which may be attributed to its characteristics as a serine protease with broad substrate specificity and diverse cleavage sites, enabling efficient cleavage of peptide bonds in PBSB (Xiang Wang et al. 2023). However, it should be noted that the commercial proteases used in this study differed in declared enzyme activity units and catalytic characteristics, which may have influenced the hydrolysis efficiency and peptide profiles obtained. Therefore, the present comparison mainly served as a preliminary screening of suitable proteases under practical application conditions.

Notably, hydrolysates with higher DH values did not always exhibit stronger α‐glucosidase inhibitory activity. This phenomenon was particularly evident in the papain‐treated group: although papain resulted in the lowest DH, its α‐glucosidase inhibitory activity was higher than that of flavor protease. This discrepancy suggests that the bioactivity of hydrolysates is not solely dependent on the extent of protein hydrolysis, but is also closely related to peptide sequence composition and structural characteristics. Specifically, peptides with suitable molecular weight distributions, hydrophobic properties, and characteristic amino acid residues are considered more favorable for α‐glucosidase inhibition (Mohd Rodhi et al. 2023; Mu et al. 2023). Papain may preferentially cleave peptide bonds that preserve or release peptide fragments with higher bioactivity, despite its relatively low hydrolysis efficiency (Sheng et al. 2023). These findings indicate that DH alone cannot fully predict the hypoglycemic potential of protein hydrolysates, and further peptide profiling analysis is necessary to clarify the structure–activity relationship of PBSBPs. Therefore, the selection of the optimal protease should comprehensively consider both hydrolysis efficiency and biological activity. Based on these findings, alkaline protease was selected as the most suitable protease under the present experimental conditions for subsequent process optimization.

#### 3.1.2 Single‐Factor Experiments

**FIGURE 2:**
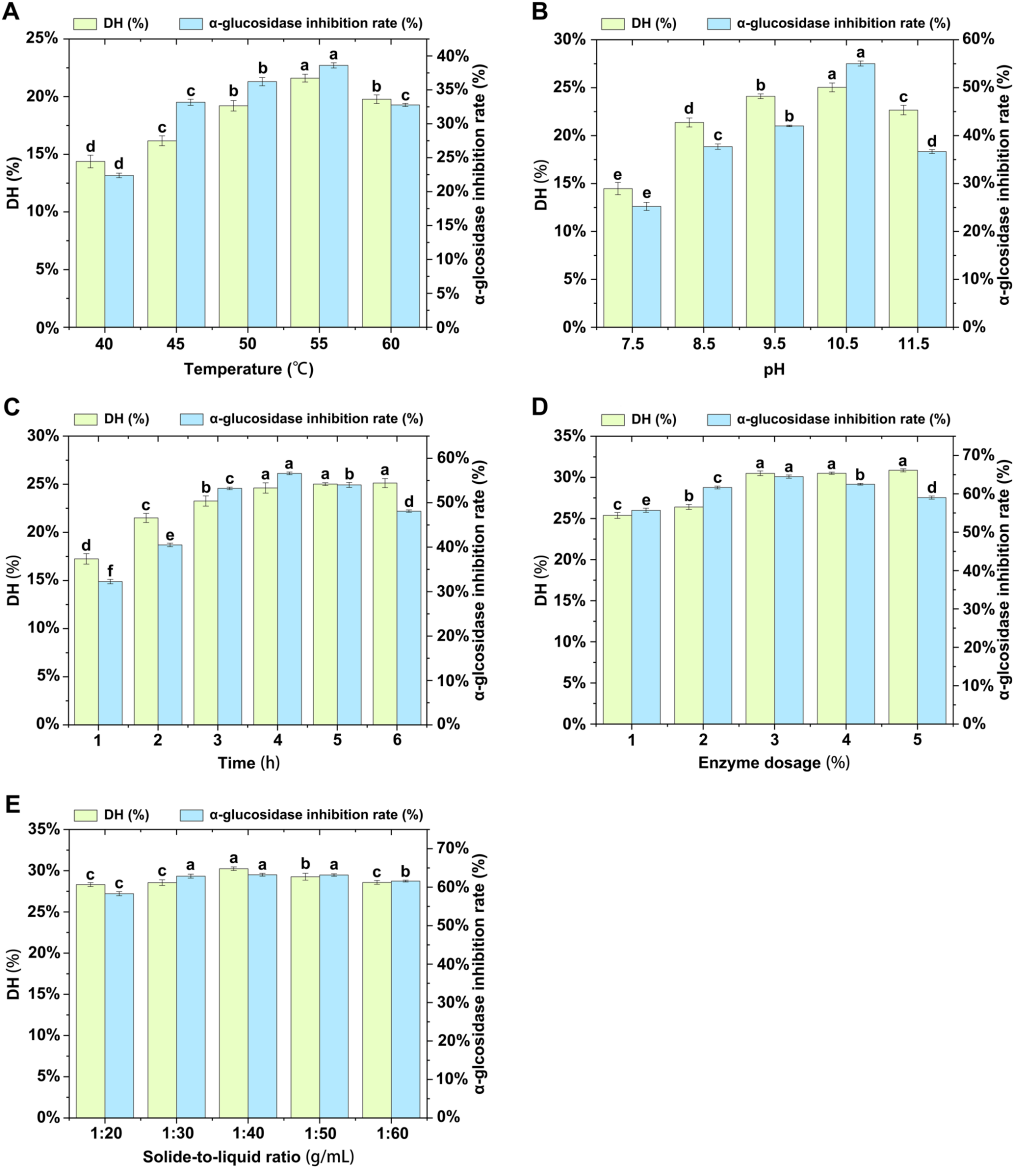
Effects of single‐factor conditions on hydrolysis degree and α‐glucosidase inhibition activity. (A) Hydrolysis temperature; (B) Hydrolysis pH; (C) Hydrolysis time; (D) Enzyme dosage; (E) Solid‐to‐liquid ratio. Values are presented as mean ± SD. Different lowercase letters in the same column indicate significant differences at *p* < 0.05 (*n* = 3). DH, hydrolysis degree.

In the enzymatic hydrolysis of PBSB, multiple factors were found to significantly affect both the DH and the α‐glucosidase inhibitory activity of the hydrolysates. As shown in Figure 2A, with increasing temperature, both the DH and α‐glucosidase inhibition rate gradually increased and reached their maximum values at 55°C. Above 55°C, both indicators decreased progressively. This trend may be attributed to the dual effects of temperature on enzyme activity: moderate heating accelerates molecular motion and enhances enzyme–substrate interactions, whereas temperatures exceeding the optimal range may induce denaturation of alkaline protease, thereby disrupting its tertiary structure and reducing catalytic efficiency (Daniel et al. 2009).

The optimal pH for both hydrolysis efficiency and α‐glucosidase inhibitory activity was 10.5 (Figure 2B). Deviations from this pH resulted in significant reductions in both DH and inhibitory activity. This phenomenon may be associated with structural alterations in the active center of the enzyme under non‐optimal pH conditions, thereby weakening enzyme–substrate binding affinity and reducing catalytic efficiency (Mora and Toldrá 2023). It should be noted that the pH associated with the highest bioactivity may not necessarily correspond exactly to the theoretically optimal catalytic pH of the enzyme. In addition to affecting enzymatic activity, pH can also influence substrate conformation, protease cleavage specificity, peptide release patterns, and the stability of generated peptide sequences (Barbosa da Silva et al. 2025; Butré et al. 2015). Therefore, the enhanced α‐glucosidase inhibitory activity observed at pH 10.5 may be related not only to improved hydrolysis efficiency, but also to the preferential generation or preservation of peptide fragments with stronger hypoglycemic activity. Similar phenomena have been reported in previous studies on bioactive peptide production, where optimal bioactivity and maximal DH did not completely coincide due to differences in peptide composition and structure (Moreno‐Mariscal et al. 2025; Wang et al. 2020).

As shown in Figure 2C, the DH gradually increased with prolonged hydrolysis time and reached a plateau after 4 h. Meanwhile, the α‐glucosidase inhibition rate reached its maximum at 4 h and then gradually decreased. This result suggests that excessive hydrolysis may further degrade bioactive peptide sequences into smaller fragments with reduced or no inhibitory activity, despite the continued increase in DH (Yin et al. 2021).

When the enzyme dosage was low, both DH and α‐glucosidase inhibitory activity remained at relatively low levels. With increasing enzyme dosage, the DH increased rapidly before gradually reaching a plateau (Figure 2D). This is probably because excessive enzyme loading fails to further enhance hydrolysis efficiency upon substrate saturation at a fixed substrate concentration. Meanwhile, the α‐glucosidase inhibition rate reached its highest value at an enzyme dosage of 3% and then gradually decreased at higher dosages. This finding further indicates that the generation of α‐glucosidase inhibitory peptides depends not only on hydrolysis efficiency but also on the preservation of specific peptide structures associated with bioactivity. Excessive enzyme dosage may lead to over‐hydrolysis of active peptides into smaller fragments with lower biological activity (Hao et al. 2024).

When the solid‐to‐liquid ratio increased from 1:20 to 1:40 g/mL, both the DH and α‐glucosidase inhibition rate gradually increased (Figure 2E). However, when the ratio further increased from 1:40 to 1:60 g/mL, both indicators showed a decreasing trend. An appropriate solid‐to‐liquid ratio may facilitate sufficient enzyme–substrate interactions and improve mass transfer efficiency during hydrolysis, thereby promoting the release of bioactive peptides (Mora and Toldrá 2023). In contrast, excessively high liquid volumes may reduce substrate concentration and reaction driving force, ultimately decreasing hydrolysis efficiency and inhibitory activity (Zhu et al. 2025).

#### 3.1.3 Results of Orthogonal Experiments

Based on the results of the single‐factor experiments, an L_9_ (3^4^) orthogonal design was employed to evaluate the effects of hydrolysis temperature (A), hydrolysis pH (B), hydrolysis time (C), and enzyme dosage (D) on the α‐glucosidase inhibitory activity of PBSBPs. The results are presented in Table 3. According to the R value analysis, the order of influence of the investigated factors was as follows: hydrolysis pH>enzyme dosage>hydrolysis time>hydrolysis temperature, indicating that pH exerted the greatest effect on α‐glucosidase inhibitory activity, whereas temperature showed the least influence.

**TABLE 3:** Results of orthogonal experiments.

Experiment No.	(A) Temperature/°C	(B) pH	(C) Time/h	(D) Enzyme dosage/%	α‐glucosidase inhibition rate/%
1	50	9.5	3	2	64.35 ± 0.32
2	50	10.5	4	3	65.45 ± 0.30
3	50	11.5	5	4	51.34 ± 0.18
4	55	9.5	4	4	61.54 ± 0.14
5	55	10.5	5	2	60.53 ± 0.33
6	55	11.5	3	3	51.35 ± 0.31
7	60	9.5	5	3	61.15 ± 0.12
8	60	10.5	3	4	57.74 ± 0.20
9	60	11.5	4	2	54.51 ± 0.13
K1	181.14	187.04	173.44	179.39	
K2	173.42	183.72	181.50	177.95	
K3	173.40	157.20	173.02	170.62	
R	2.58	9.95	2.83	2.93	
k1	60.38	62.35	57.81	59.80	
k2	57.81	61.24	60.50	59.32	
k3	57.80	52.40	57.67	56.87	
Optimal combination	A_1_B_1_C_2_D_1_				

*Note:* values are presented as mean ± SD (*n* = 3).

Further analysis of the *K*‐values indicated that the optimal hydrolysis condition combination was A_1_B_1_C_2_D_1_, corresponding to a hydrolysis temperature of 50°C, pH 9.5, hydrolysis time of 4 h, and enzyme dosage of 2%. Under these optimized conditions, the α‐glucosidase inhibition rate of PBSBPs reached 67.90% ± 0.26% at 5 mg/mL. Compared with the preliminary protease screening results, the inhibitory activity was further improved after process optimization, indicating that appropriate hydrolysis conditions are critical for the generation and preservation of bioactive peptide sequences.

According to the orthogonal experimental results, the maximum α‐glucosidase inhibitory activity of PBSBPs was achieved under the hydrolysis condition combination A_1_B_1_C_2_D_1_. Therefore, all PBSBPs used in the subsequent physicochemical characterization and animal experiments were prepared under these optimized conditions.

### 3.2 Analysis of Activity and Physicochemical Properties of Peptides

Previous studies have demonstrated a strong correlation between peptide bioactivity and PeptideRanker scores (Pekkoh et al. 2025). To further investigate the sequence–bioactivity relationship of PBSBPs and clarify the possible structural basis underlying their hypoglycemic activity, peptide sequences in the hydrolysates were identified using Nano‐LC–MS/MS and subsequently analyzed using online bioinformatics platforms. Nano‐LC–MS/MS was employed because it enables high‐sensitivity identification of low‐molecular‐weight peptides in complex hydrolysates, thereby facilitating subsequent structure–activity relationship analysis (Xie and Butler 2025). Peptides with PeptideRanker scores higher than 0.5 are generally considered to possess potential biological activity (W. Zheng et al. 2025). Based on this criterion, a total of 101 peptide sequences were identified from PBSBPs, and most peptides exhibited scores above 0.5, indicating considerable bioactivity potential.

**FIGURE 3:**
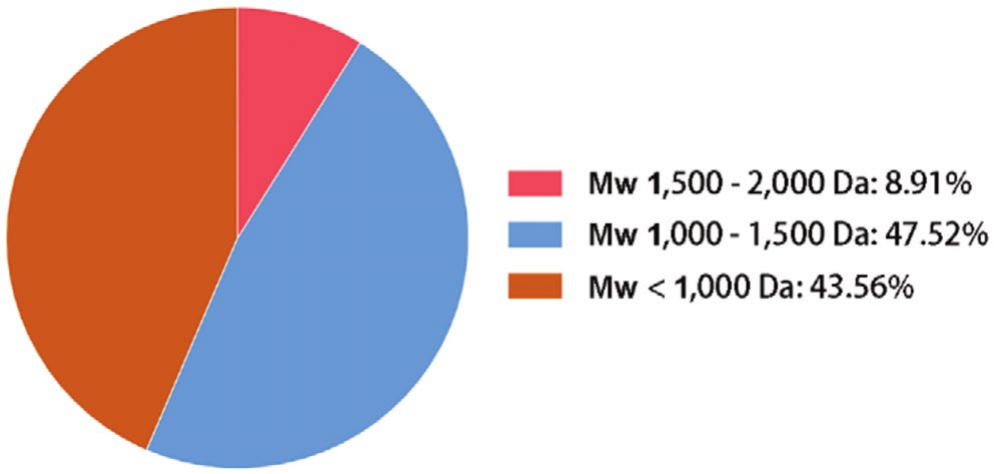
Molecular weight distribution of 101 identified peptides from PBSBPs.

Previous studies have shown that the α‐glucosidase inhibitory activity of peptides is closely associated with molecular weight, hydrophobicity, and amino acid composition (Y. Zhang et al. 2017). In the present study, 91.09% of the identified peptides had molecular weights below 1500 Da (Figure 3), which is consistent with the broad substrate specificity of alkaline protease that preferentially generates small peptide fragments. These low‐molecular‐weight peptides are generally considered more favorable for accessing the active site of α‐glucosidase and may exhibit improved gastrointestinal stability, thereby contributing to enhanced inhibitory activity.

Furthermore, hydrophobic and branched‐chain amino acid residues such as leucine (L), valine (V), phenylalanine (F), proline (P), alanine (A), and arginine (R) are frequently found in peptides exhibiting α‐glucosidase inhibitory activity (Cian et al. 2022; Maestri et al. 2019). The identified peptides in PBSBPs contained relatively high proportions of these characteristic amino acid residues. Previous studies have also suggested that peptides containing hydrophobic residues at the C‐terminus may exhibit enhanced hypoglycemic activity (Nongonierma and FitzGerald 2019). In the present study, 19 identified peptides possessed alanine residues at the C‐terminus, which may contribute to α‐glucosidase inhibitory activity, possibly due to enhanced peptide stability and favorable enzyme–peptide interactions.

To further screen peptides with potential hypoglycemic activity, the BIOPEP‐UWM database was employed for functional prediction (Minkiewicz et al. 2019). A total of 58 peptides were predicted to possess potential α‐glucosidase inhibitory activity, including GAPGPSGPPGPA and GAAGPPGPVGPG. In addition, the isoelectric point (pI) values of these peptides were calculated using the ProtParam tool available on ExPASy (Wilkins et al. 1999). Detailed information for all identified peptides is listed in Table S1.

Collectively, the predominance of low‐molecular‐weight peptides enriched in hydrophobic amino acid residues may contribute to the observed α‐glucosidase inhibitory activity of PBSBPs, since these structural characteristics are considered favorable for interactions with the hydrophobic regions surrounding the catalytic site of α‐glucosidase.

While the present study mainly focused on the hypoglycemic activity of PBSBPs, comprehensive nutritional evaluation, including amino acid composition analysis and nutritional indices such as Protein Efficiency Ratio (PER) and Chemical Score, was not conducted. These aspects remain important directions for future studies to further assess the nutritional and functional value of PBSB‐derived peptides.

### 3.3 Animal Experiments

#### 3.3.1 Effect of PBSBPs on Glucose Tolerance in Mice

**FIGURE 4:**
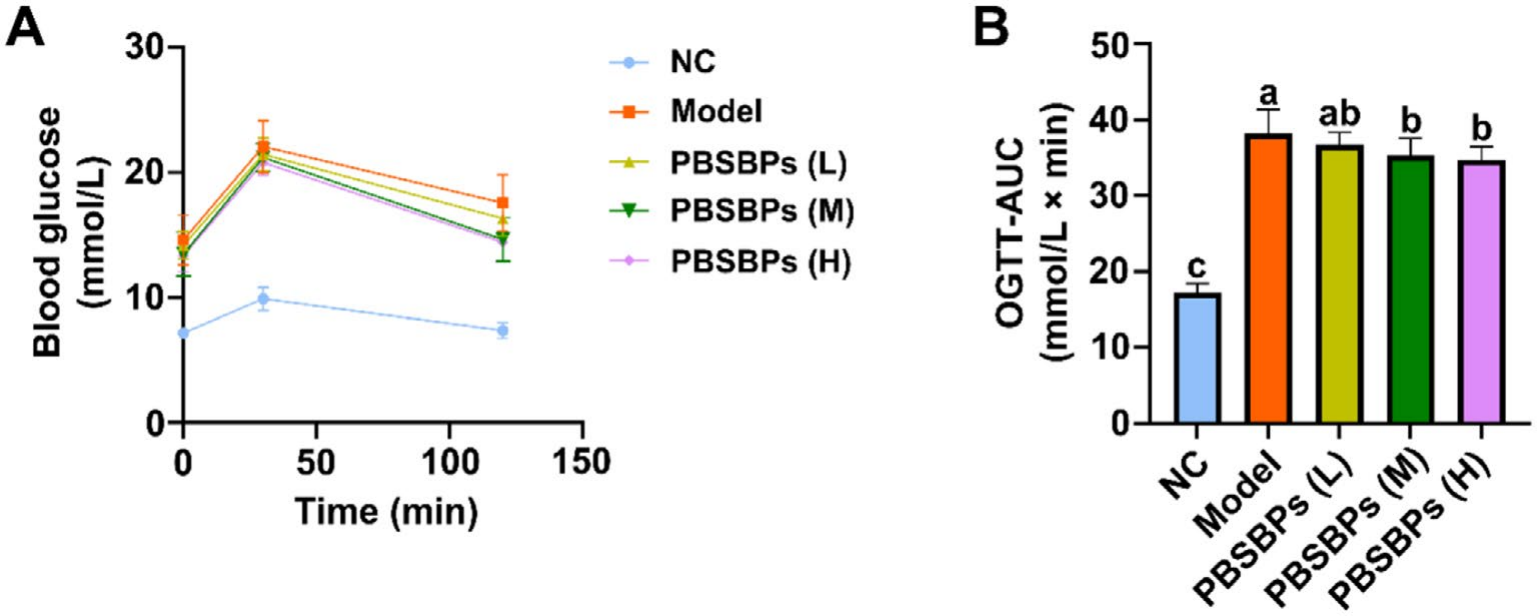
Effects of PBSBPs intervention on blood glucose regulation in mice. (A) OGTT results and (B) Area under the curve (AUC). Values are presented as mean ± SD. Different lowercase letters indicate significant differences (*p* < 0.05, *n = 10). OGTT ral glucose toleran*ce test; NC, normal control; PBSB *P. bocourti* swim bladder peptides; PBSBPs (L): low‐dose PBSBPs group; PBSBPs (M): middle‐dose PBSBPs group; PBSBPs (H): high‐dose PBSBPs group.

Changes in glucose tolerance in mice provide preliminary evidence for the in vivo hypoglycemic activity of PBSBPs. As shown in Figure 4A, blood glucose levels in all groups reached peak values at 30 min after glucose gavage and then gradually declined over time. However, compared with the NC group, mice in the Model group maintained significantly higher blood glucose levels at 120 min (*p* < 0.05), indicating impaired glucose tolerance. This impairment may be associated with insulin resistance, which is characterized by reduced insulin sensitivity in liver, muscle, and adipose tissues, thereby interfering with glucose uptake and utilization (Echouffo‐Tcheugui and Selvin 2021).

Further analysis demonstrated that the AUC values in the PBSBPs (L), PBSBPs (M), and PBSBPs (H) groups were reduced by 3.79%, 7.55%, and 9.02%, respectively, compared with the Model group (*p* < 0.05) (Figure 4B), suggesting that PBSBPs improved glucose tolerance in diabetic mice. This improvement may be associated with the α‐glucosidase inhibitory activity of PBSBPs observed in vitro. By delaying carbohydrate digestion and glucose absorption in the intestine, PBSBPs may reduce postprandial blood glucose fluctuations and alleviate the metabolic burden on pancreatic β‐cells (Li et al. 2025). However, additional mechanistic studies are still required to further clarify the molecular pathways underlying these hypoglycemic effects.

#### 3.3.2 Effects of PBSBPs on Blood Lipid Profiles

Type 2 diabetes mellitus is frequently accompanied by disorders of lipid metabolism (Long et al. 2021). Such dyslipidemia is closely associated with impaired glucose metabolism and may contribute to excessive lipid accumulation in diabetic mice. Alterations in serum lipid profiles therefore provide additional evidence for evaluating the in vivo metabolic regulatory effects of PBSBPs.

**FIGURE 5:**
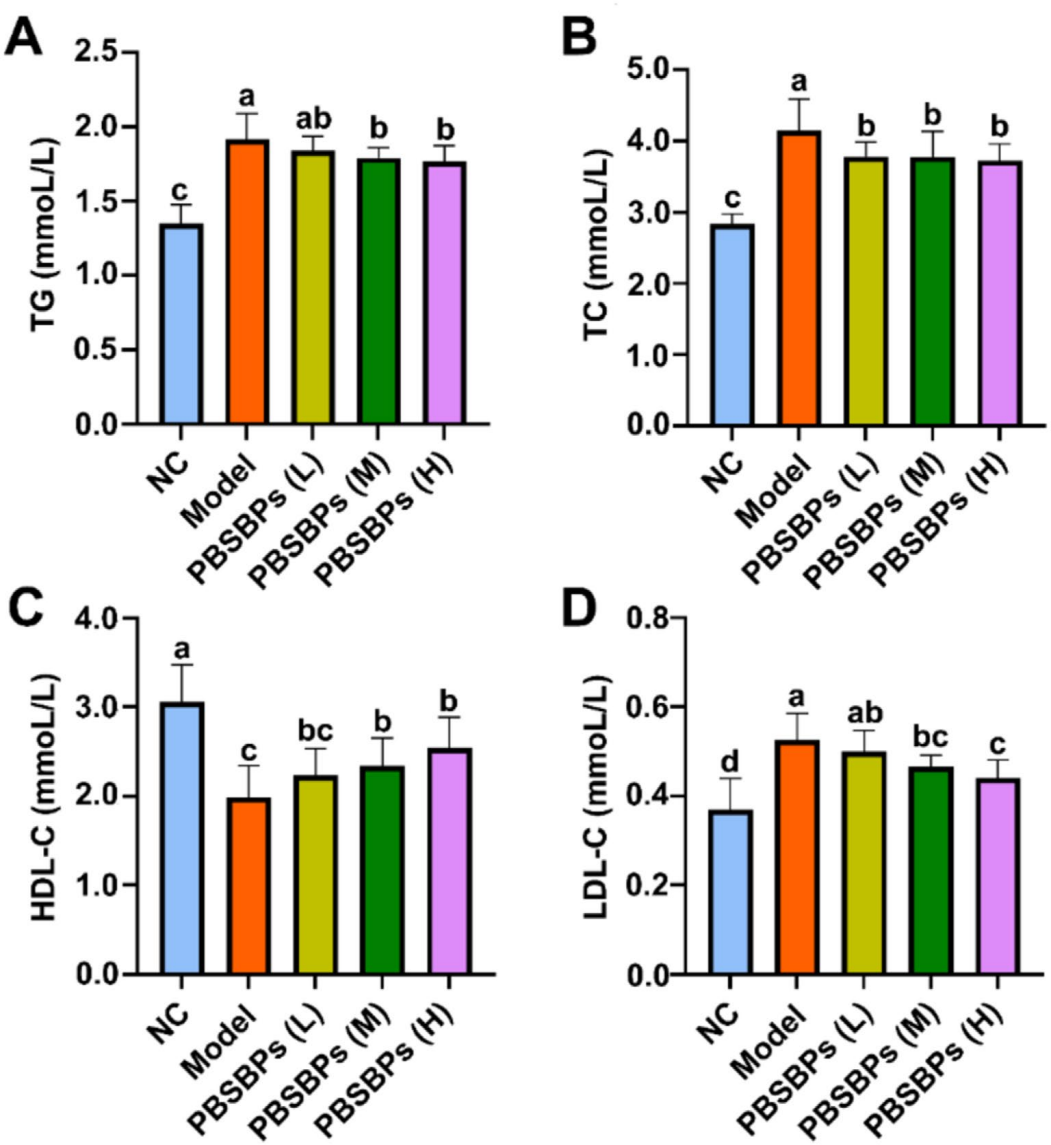
Effects of PBSBPs on serum lipid profiles in diabetic mice. (A) TG; (B) TC; (C) HDL‐C; (D) LDL‐C. Values are presented as mean ± SD. Different lowercase letters indicate significant differences (*p* < 0.05, *n* = 1*0*). HDL‐C, high‐density lipoprotein cholesterol; LDL‐C, low‐density lipoprotein cholesterol; NC, normal control; PBSBPs (H), high‐dose group; PBSBPs (L), low‐dose group; PBSBPs (M), medium‐dose group; PBSBPs, *P. bocourti* swim bladder peptides; TC, total cholesterol; TG, triglycerides.

As shown in Figure 5, the levels of TG (Figure 5A), TC (Figure 5B), and LDL‐C (Figure 5D) in the Model group were significantly higher than those in the NC group (*p* < 0.05), which is consistent with the typical dyslipidemic characteristics observed in type 2 diabetic mice. In contrast, all PBSBPs‐treated groups exhibited lower TG, TC, and LDL‐C levels compared with the Model group, with the most pronounced improvement observed in the high‐dose group (*p* < 0.05). These results suggest that PBSBPs may alleviate lipid metabolic disorders, possibly through modulation of glucose and lipid metabolic homeostasis.

Additionally, the HDL‐C level in the Model group was significantly lower than that in the NC group (*p* < 0.05) (Figure 5C). Elevated HDL‐C levels are generally considered beneficial for reverse cholesterol transport and the maintenance of lipid homeostasis (von Eckardstein et al. 2023). Notably, HDL‐C levels in PBSBPs‐treated groups increased gradually with increasing PBSBPs dosage compared with the Model group (*p* < 0.05). Collectively, these findings indicate that PBSBPs can improve blood lipid profiles in diabetic mice and may contribute to reducing the risk of metabolic complications associated with diabetes (Xuan et al. 2023).

#### 3.3.3 H&E Staining for Histological Analysis

**FIGURE 6:**
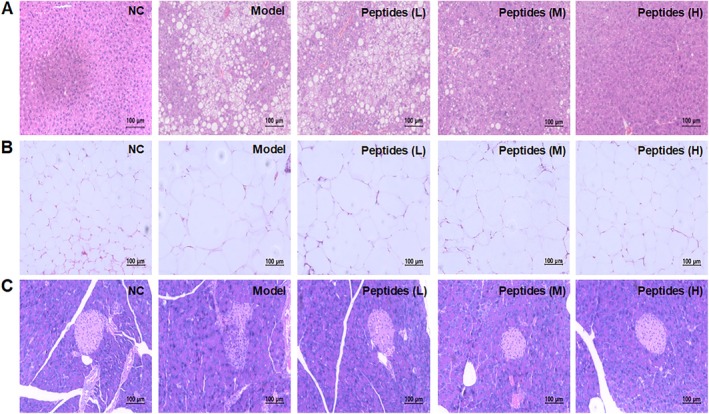
Histopathological observation of major tissues in mice. (A) Liver tissue; (B) Epididymal adipose tissue; (C) Pancreatic tissue. Scale bar = 100 μm. NC, normal control; PBSBPs (H), high‐dose group; PBSBPs (L), low‐dose group; PBSBPs (M), medium‐dose group; PBSBPs, *P. bocourti* swim bladder peptides.

As shown in Figure 6A, hepatocytes in the NC group exhibited normal morphology without obvious lipid droplet vacuoles. In contrast, hepatocytes in the Model group displayed disordered cellular morphology and abundant intracellular lipid droplet vacuoles of different sizes, indicating hepatic steatosis. Following PBSBPs intervention, the number of hepatic lipid droplet vacuoles was markedly reduced. These findings suggest that PBSBPs may alleviate hepatic lipid accumulation and improve hepatic steatosis in diabetic mice. This improvement is likely associated with the amelioration of glucose and lipid metabolic imbalance (Leclercq et al. 2007; Zhong et al. 2024).

As shown in Figure 6B, epididymal adipocytes in the NC group displayed normal morphology, whereas adipocytes in the Model group showed obvious hypertrophy. In contrast, adipocyte size in the PBSBPs‐treated groups was significantly reduced compared with the Model group. This result suggests that PBSBPs may improve metabolic status in diabetic mice by alleviating adipocyte hypertrophy. Previous studies have indicated that smaller adipocytes tend to exhibit superior metabolic flexibility, higher insulin responsiveness, and greater capacity for glucose uptake and storage (McLaughlin et al. 2016).

Additionally, PBSBPs intervention significantly improved pancreatic islet morphology. As shown in Figure 6C, islets in the Model group exhibited pathological changes including irregular morphology and blurred boundaries, which are characteristic features of pancreatic injury induced by long‐term high‐fat and high‐sugar diets (Han et al. 2023). In contrast, islets in PBSBPs‐treated groups exhibited more regular morphology and clearer boundaries. Collectively, these results indicate that PBSBPs can reduce hepatic lipid accumulation, alleviate adipocyte hypertrophy, and improve pancreatic islet morphology in diabetic mice, suggesting their beneficial effects on metabolic disorders associated with diabetes.

## 4 Conclusion

In this study, alkaline protease produced the most favorable hydrolysate under the present experimental conditions for the hydrolysis of *Pangasius bocourti* swim bladder (PBSB). The optimized hydrolysis conditions were determined as follows: temperature 50°C, pH 9.5, hydrolysis time 4 h, and enzyme dosage 2% (w/w). Under these conditions, the obtained PBSBPs exhibited an α‐glucosidase inhibition rate of 67.90% ± 0.26% at 5 mg/mL, indicating notable in vitro α‐glucosidase inhibitory activity. Nano‐LC–MS/MS analysis identified 101 peptides, among which 91.09% possessed molecular weights below 1500 Da. BIOPEP‐UWM analysis further predicted 58 peptides with potential α‐glucosidase inhibitory activity. The predominance of low‐molecular‐weight peptides enriched in hydrophobic amino acid residues may contribute to the observed hypoglycemic activity of PBSBPs. In vivo experiments demonstrated that PBSBPs significantly improved glucose tolerance and blood lipid profiles, alleviated hepatic steatosis, reduced epididymal adipocyte hypertrophy, and improved pancreatic islet morphology in diabetic mice.

Nevertheless, several limitations remain in the present study. The structure–activity relationships of the identified peptides have not yet been systematically clarified, and their gastrointestinal stability, bioavailability, and precise molecular mechanisms of action remain unclear. In particular, the interactions between specific peptides and α‐glucosidase, as well as their potential regulatory effects on glucose metabolism‐related signaling pathways, require further investigation. Therefore, future studies should focus on (i) systematic structure–activity relationship analysis of the identified peptides, (ii) evaluation of digestive stability and bioavailability, (iii) mechanistic studies at the molecular and cellular levels, and (iv) long‐term safety and efficacy assessments in animal models. Addressing these aspects will facilitate the development of PBSBPs as natural hypoglycemic functional ingredients and promote the sustainable valorization of aquatic processing by‐products.

## Author Contributions

**Kefeng Wu:** conceptualization, validation, writing – review and editing. **Yushuang Li:** methodology, formal analysis, validation. **Junde Chen:** methodology, validation, writing – review and editing, resources, funding acquisition. **Huan Zhang:** conceptualization, methodology, data curation, validation, formal analysis, writing – original draft. **Xixiang Tang:** project administration, resources, writing – review and editing, funding acquisition. **Guangxin Xu:** methodology, validation, data curation. **Xu Qiu:** formal analysis, validation, visualization, writing – review and editing, funding acquisition. **Jianlin He:** methodology, validation, writing – original draft, resources. **Ju Yang:** conceptualization, methodology, data curation, software, writing – original draft, visualization.

## Funding

This work was supported by Innovation Research and Development Special Funds of the Municipality‐Province‐Ministry Co‐constructed, GJZX‐HYSW‐2025‐03, GJZX‐HYSW‐2024‐01. Scientific Research Foundation of Third Institute of Oceanography, MNR, 2026018. Fujian Provincial Science and Technology Program Project, 2025Y0081.

## Ethics Statement

The animal experiments were conducted in agreement with the UK Animals (Scientific Procedures) Act 1986 and the relevant guidelines. The animal experiments in this study (ethical approval number: TIO‐IACUC‐01‐2024‐03‐11) were evaluated and approved by the Third Institute of Oceanography, Ministry of Natural Resources.

## Conflicts of Interest

The authors declare no conflicts of interest.

## Supporting information


**Table S1:** The sequences, physicochemical properties, and bioactivities of peptides.

## Data Availability

The data that support the findings of this study are available from the corresponding author upon reasonable request.
