# Supplementary material for: Preparation and Hypoglycemic Activity of Enzymatically Hydrolyzed Peptides From the Swim Bladder of Pangasius bocourti
Source: Food Sci Nutr. 2026 Jul 1;14(7):e72061. doi: 10.1002/fsn3.72061 (PMC13322661; doi:10.1002/fsn3.72061)
Supplement: Supplementary file 1 — Table S1: The sequences, physicochemical properties, and bioactivities of peptides. [file FSN3-14-e72061-s001.docx]

**Table S1. The sequences, physicochemical properties, and bioactivities of peptides**

| **NO.** | **Peptide sequence** | **Length** | **Molecular weight (Da)** | **Bioactivity Ranker** | **Prediction of biological activity by BIOPEP-UWM** | **pI** |
| --- | --- | --- | --- | --- | --- | --- |
|  |  |  |  |  | **α-glucosidase** |  |
| 1 | DSHPPRLF | 8 | 967.4875 | 0.89988 | + | 6.74 |
| 2 | GPIGYPGPR | 9 | 928.4766 | 0.897279 | + | 8.75 |
| 3 | GGPPGPTGPR | 10 | 907.4511 | 0.880336 | + | 9.75 |
| 4 | GAPGPSGPPGPA | 12 | 992.4562 | 0.814343 | + | 5.52 |
| 5 | GAAGPPGPVGPG | 12 | 948.4664 | 0.875857 | + | 5.52 |
| 6 | ADPFHGYR | 8 | 961.4406 | 0.874694 | + | 6.79 |
| 7 | ADPFHGY | 7 | 805.3395 | 0.826696 | + | 5.08 |
| 8 | PVGPPGPR | 8 | 791.429 | 0.801711 | + | 10.18 |
| 9 | GERGPPGPM | 9 | 912.4123 | 0.769878 | + | 6.00 |
| 10 | TGPVGPPGLR | 10 | 965.5294 | 0.692781 | + | 9.41 |
| 11 | GRVGPPGPAGA | 11 | 950.4933 | 0.585778 | + | 9.75 |
| 12 | AGRLGPPGPA | 10 | 907.4875 | 0.558128 | + | 9.79 |
| 13 | GRVGPPGPA | 10 | 893.4719 | 0.535423 | + | 9.75 |
| 14 | GRLGPPGPA | 10 | 907.4875 | 0.586217 | + | 9.75 |
| 15 | TGPIGPPGPAG | 11 | 935.4712 | 0.578415 | + | 5.19 |
| 16 | GPPGPPGPPGPPGPS | 15 | 1311.6094 | 0.949508 | + | 5.52 |
| 17 | GPRGPPGPPGL | 11 | 1016.5403 | 0.940988 | + | 9.75 |
| 18 | GPPGPVGPGGK | 11 | 934.4872 | 0.915637 | + | 8.75 |
| 19 | GPAGPPGFPGGPGAK | 15 | 1310.6254 | 0.905763 | + | 8.75 |
| 20 | GPGGPSGERGPPGPM | 15 | 1348.6194 | 0.903879 | + | 6.0 |

**Table S1 (Continued). The sequences, physicochemical properties, and bioactivities of peptides**

| **NO.** | **Peptide sequence** | **Length** | **Molecular weight (Da)** | **Bioactivity Ranker** | **Prediction of biological activity by BIOPEP-UWM** | **pI** |
| --- | --- | --- | --- | --- | --- | --- |
|  |  |  |  |  | **α-glucosidase** |  |
| 21 | GPRGPPGPPGLS | 12 | 1087.5774 | 0.883426 | + | 9.75 |
| 22 | GLPGPIGPPGPR | 12 | 1113.6294 | 0.881161 | + | 9.75 |
| 23 | GIPGPIGPPGPR | 12 | 1113.6294 | 0.881148 | + | 9.75 |
| 24 | PAGPPGFPGGPGAK | 14 | 1253.6039 | 0.879191 | + | 9.18 |
| 25 | GIPGPVGPPGPR | 12 | 1131.6035 | 0.863358 | + | 9.75 |
| 26 | SPGPAGPSGPSGPPGAR | 18 | 1533.717 | 0.862856 | + | 9.47 |
| 27 | GPSGERGPPGPM | 12 | 1153.5186 | 0.861882 | + | 6.00 |
| 28 | GPSGPPGPDGNKGEPG | 18 | 1576.7117 | 0.857023 | + | 4.37 |
| 29 | GPPGPPGLSGPQ | 12 | 1059.5349 | 0.853165 | + | 5.52 |
| 30 | GPSGPSGPPGAR | 12 | 1051.5046 | 0.846985 | + | 9.75 |
| 31 | GPPGPVGPGGKEGQRG | 17 | 1532.7694 | 0.845133 | + | 8.75 |
| 32 | GPPGFAGPPGADGQSGAK | 18 | 1614.7273 | 0.841881 | + | 5.84 |
| 33 | GPRGPPGPPGLSGPQ | 15 | 1401.7 | 0.838299 | + | 9.75 |
| 34 | GPDGAPGKDGLR | 12 | 1138.573 | 0.836732 | + | 5..96 |
| 35 | GPVGLPGPPGPPGPQ | 15 | 1370.6829 | 0.830144 | + | 5.52 |
| 36 | GLIGPPGPSGERGAPGESGAK | 21 | 1921.9492 | 0.828955 | + | 6.14 |
| 37 | GPVGPRGPPGPPGKA | 15 | 1371.7258 | 0.820092 | + | 11.00 |
| 38 | GPRGPIGPPGRNGDDGEAGKA | 21 | 1990.9456 | 0.819821 | + | 6.12 |
| 39 | GRVGPPGAP | 9 | 822.4348 | 0.48642 | + | 9.75 |
| 40 | WSDLHPGAPQVK | 12 | 1333.6779 | 0.420789 | + | 6.74 |
| 41 | ILPEMGETPLFK | 12 | 1373.7264 | 0.399266 | + | 4.53 |

**Table S1 (Continued). The sequences, physicochemical properties, and bioactivities of peptides**

| **NO.** | **Peptide sequence** | **Length** | **Molecular weight (Da)** | **Bioactivity Ranker** | **Prediction of biological activity by BIOPEP-UWM** | **pI** |
| --- | --- | --- | --- | --- | --- | --- |
|  |  |  |  |  | **α-glucosidase** |  |
| 42 | VEGPDFDVTLPKA | 13 | 1386.703 | 0.381106 | + | 4.03 |
| 43 | DTGAPIRIPVGPE | 15 | 1534.8354 | 0.307161 | + | 4.37 |
| 44 | SVEYIPYEPGTY | 12 | 1416.6449 | 0.304027 | + | 3.79 |
| 45 | GPPGFAGPPGADGQ | 14 | 1255.5469 | 0.784047 | + | 3.80 |
| 46 | IKIIAPPERKY | 11 | 1326.8022 | 0.302725 | + | 9.70 |
| 47 | GPRGPSGPPGAPGPQ | 15 | 1343.6581 | 0.762962 | + | 9.75 |
| 48 | RPGPPGPVGAR | 11 | 1091.5835 | 0.761061 | + | 12.00 |
| 49 | GPAGQDGAAGPPGPA | 15 | 1234.5577 | 0.758612 | + | 3.8 |
| 50 | GPPGLAGPPGEAGRE | 15 | 1376.6683 | 0.792992 | + | 4.53 |
| 51 | GPVGPPGNPGAN | 12 | 1064.4886 | 0.738577 | + | 5.52 |
| 52 | GPAGDSGRPGEAGLVGAR | 18 | 1638.8073 | 0.730613 | + | 6.07 |
| 53 | GPPGPVGPGGKEGQ | 10 | 1032.5352 | 0.697384 | + | 6.0 |
| 54 | GPPGSTGPAGKDGPRGPRGDVGPA | 24 | 2172.0671 | 0.718635 | + | 8.75 |
| 55 | GPRGPPGPSGSPGPQ | 15 | 1375.6479 | 0.706422 | + | 9.75 |
| 56 | GEPGPSGPQ | 9 | 824.3664 | 0.390873 | - | 4.0 |
| 57 | GTPGFPGPR | 9 | 916.4402 | 0.897607 | - | 9.75 |
| 58 | GSVGAVGPK | 9 | 770.4286 | 0.310452 | - | 8.75 |
| 59 | GESGYRGL | 8 | 837.3981 | 0.541123 | - | 6.0 |
| 60 | GDVGPTGPAGPA | 12 | 994.4719 | 0.310452 | - | 3.8 |
| 61 | GGYDEYR | 7 | 858.3508 | 0.404356 | - | 4.37 |
| 62 | GERGTPGGIGA | 11 | 986.4781 | 0.313339 | - | 6.00 |

**Table S1 (Continued). The sequences, physicochemical properties, and bioactivities of peptides**

| **NO.** | **Peptide sequence** | **Length** | **Molecular weight (Da)** | **Bioactivity Ranker** | **Prediction of biological activity by BIOPEP-UWM** | **pI** |
| --- | --- | --- | --- | --- | --- | --- |
|  |  |  |  |  | **α-glucosidase** |  |
| 63 | GRTGEIGPA | 9 | 856.4402 | 0.195243 | - | 6.00 |
| 64 | TGEQGSVGPA | 10 | 901.4141 | 0.108175 | - | 4.00 |
| 65 | GLPGERGRVGPS | 12 | 1196.6261 | 0.352592 | - | 9.60 |
| 66 | VIDDHYYHY | 9 | 1223.5247 | 0.379123 | - | 5.05 |
| 67 | GERGEQGPAGSPGFQ | 15 | 1488.6593 | 0.402793 | - | 4.53 |
| 68 | RGIEGPVGAV | 10 | 953.5294 | 0.163462 | - | 6.00 |
| 69 | RYDKDLSDRIF | 11 | 1426.7205 | 0.401607 | - | 6.04 |
| 70 | KVPQPAEGEGELELK | 15 | 1622.8514 | 0.398446 | - | 4.49 |
| 71 | FERDLQGR | 9 | 1019.5148 | 0.3787 | - | 6.07 |
| 72 | IDGGDAVDELDFK | 13 | 1392.6409 | 0.35038 | - | 3.71 |
| 73 | GPVGVTGPK | 9 | 810.4599 | 0.335803 | - | 8.75 |
| 74 | VHVGGDDFVHL | 11 | 1193.5829 | 0.355448 | - | 5.05 |
| 75 | GAPGFPGPR | 9 | 886.4296 | 0.956569 | - | 9.75 |
| 76 | GPDPGPGPM | 9 | 839.3483 | 0.944703 | - | 3.80 |
| 77 | GPVGMPGERG | 10 | 971.4494 | 0.666254 | - | 6.0 |
| 78 | GPVGMPGER | 9 | 914.428 | 0.666254 | - | 6.0 |
| 79 | PSGPRGPAGPS | 11 | 978.4882 | 0.624063 | - | 10.18 |
| 80 | DPGPVGVK | 8 | 783.4126 | 0.414706 | - | 5.84 |
| 81 | PGPVGVK | 7 | 652.3907 | 0.421076 | - | 9.18 |
| 82 | AAGRVGPPGPA | 11 | 964.509 | 0.4689 | + | 9.79 |
| 83 | HIPRSPFEVK | 10 | 1208.6665 | 0.408129 | - | 8.75 |

**Table S1 (Continued). The sequences, physicochemical properties, and bioactivities of peptides**

| **NO.** | **Peptide sequence** | **Length** | **Molecular weight (Da)** | **Bioactivity Ranker** | **Prediction of biological activity by BIOPEP-UWM** | **pI** |
| --- | --- | --- | --- | --- | --- | --- |
|  |  |  |  |  | **α-glucosidase** |  |
| 84 | LLPVHPRPN | 9 | 1041.6083 | 0.429561 | - | 9.76 |
| 85 | IVGFPGQK | 8 | 860.4755 | 0.370344 | - | 8.75 |
| 86 | GPAGPRGPAGPA | 12 | 1003.5199 | 0.835314 | - | 9.75 |
| 87 | GPSGPRGPAGPSGPA | 15 | 1260.6211 | 0.811236 | - | 9.75 |
| 88 | GPAGPRGPVGV | 11 | 962.5297 | 0.784578 | - | 9.75 |
| 89 | GPAGPRGPVGVA | 12 | 1033.5668 | 0.739362 | - | 9.75 |
| 90 | LTPIVEIIR | 9 | 1052.6593 | 0.130435 | + | 6.00 |
| 91 | APQEKAPDPF | 10 | 1114.5294 | 0.51924 | - | 4.37 |
| 92 | VFPGIPDHLDAA | 12 | 1250.6295 | 0.447168 | - | 4.20 |
| 93 | NWDDMEKIW | 9 | 1251.5231 | 0.680049 | - | 4.03 |
| 94 | VYRPTPN | 7 | 845.4395 | 0.306191 | - | 8.72 |
| 95 | KGPDPGPGPM | 10 | 951.4484 | 0.91699 | - | 5.84 |
| 96 | KDLAEDAPWKK | 11 | 1299.6823 | 0.410978 | - | 6.12 |
| 97 | VGPAGPRGPR | 10 | 962.541 | 0.781761 | - | 12.00 |
| 98 | FFPGEFTPEVH | 11 | 1305.6029 | 0.453023 | + | 4.51 |
| 99 | GAPGPQGPVGV | 11 | 950.4821 | 0.798964 | - | 5.52 |
| 100 | VPIPVPLPM | 9 | 977.562 | 0.831474 | - | 5.49 |
| 101 | SHNDVDKIAF | 10 | 1144.5513 | 0.396518 | - | 5.19 |
